# 1259. Successful Inpatient Penicillin Delabeling in Minimal and Low Risk Penicillin Allergic Patients

**DOI:** 10.1093/ofid/ofad500.1099

**Published:** 2023-11-27

**Authors:** Robert Petrak, Shivanjali Shankaran, Benjamin Goldenberg, Sarah Y Won, Fischer Herald, Anum Fayyaz, Hayley A Hodgson

**Affiliations:** Rush University Medical Center, Western Springs, Illinois; Rush University Medical Center, Western Springs, Illinois; Rush University Medical Center, Western Springs, Illinois; Rush University Medical Center, Western Springs, Illinois; Rush University Medical Center, Western Springs, Illinois; Rush University Medical Center, Western Springs, Illinois; Rush University Medical Center, Western Springs, Illinois

## Abstract

**Background:**

In the US, 10% of adults report a Penicillin (PCN) allergy but true IgE-mediated or serious reactions are rare. A PCN allergy label carries risk for harm including fewer first-line treatment options, increased antimicrobial resistance, toxicities, surgical site infections, and death. The objective of this study was to identify and safely remove penicillin allergy labels in eligible hospitalized patients.

**Methods:**

Between June 2022 - February 2023, the Antimicrobial Stewardship team electronically identified hospitalized patients with documented PCN allergy admitted to adult internal medicine wards at Rush University Medical Center (RUMC), an academic hospital in Chicago, IL. Patients with possible minimal or low risk PCN allergy reactions via chart review were administered a standardized questionnaire, followed by triage into Minimal, Low, and Moderate Risk PCN allergy categories (Figure 1). Minimal Risk patients were delabeled by history alone, or per patient request, after amoxicillin challenge. Low risk patients were offered an amoxicillin challenge with a single 500mg oral dose. Moderate or high risk patients were excluded.

Penicillin Allergy Risk Stratification

**Figure:**
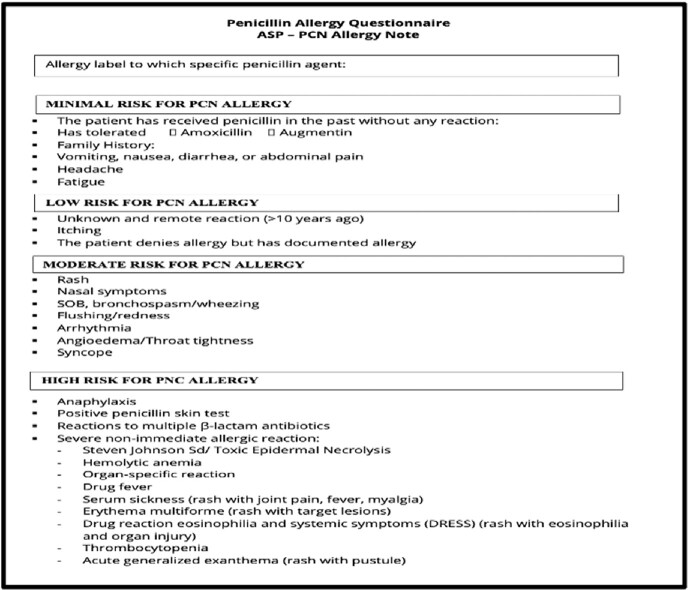

**Results:**

Of 47 interviewed patients, 31 patients (20 minimal and 11 low risk) were appropriate for delabeling. 27 of 31 (87%) were successfully delabeled via history or amoxicillin challenge; 4 were not delabeled, due to patient discomfort or early discharge. 14 of the 31 (45%) were delabeled based on history alone; all had tolerated amoxicillin before. 13 amoxicillin challenges were administered, 9 of 11 patients in low risk, 4 of 20 in minimal risk group per patient request. 100% of amoxicillin challenges were well tolerated and patients successfully delabeled. Only 1 patient was relabeled at a subsequent hospitalization.

Penicillin Delabeling Results

**Figure:**
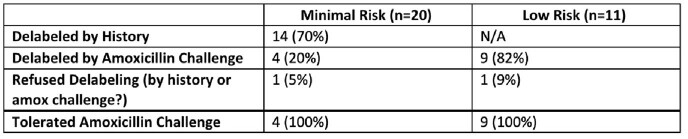

**Conclusion:**

In this proof-of-concept intervention, patients with a minimal or low risk penicillin allergy were safely delabeled as inpatients by history or amoxicillin challenge. Expanding this to a larger scale on the inpatient and outpatient settings would enable more patients with PCN allergy to be delabeled, leading to use of standard of care antibiotics and improved outcomes.

**Disclosures:**

**All Authors**: No reported disclosures

